# Temperate and tropical lizards are vulnerable to climate warming due to increased water loss and heat stress

**DOI:** 10.1098/rspb.2022.1074

**Published:** 2022-08-10

**Authors:** Chunrong Mi, Liang Ma, Yang Wang, Danyang Wu, Weiguo Du, Baojun Sun

**Affiliations:** ^1^ Key Laboratory of Animal Ecology and Conservation Biology, Institute of Zoology, Chinese Academy of Sciences, Beijing 100101, People's Republic of China; ^2^ University of Chinese Academy of Sciences, Beijing 100049, People's Republic of China; ^3^ Princeton School of Public and International Affairs, Princeton University, Princeton, NJ 08544, USA; ^4^ School of Biological Sciences, Hebei Normal University, Shijiazhuang, People's Republic of China

**Keywords:** climate warming, latitudinal vulnerability, heat tolerance, water loss, biophysical model

## Abstract

Climate warming has imposed profound impacts on species globally. Understanding the vulnerabilities of species from different latitudinal regions to warming climates is critical for biological conservation. Using five species of *Takydromus* lizards as a study system, we quantified physiological and life-history responses and geography range change across latitudes under climate warming. Using integrated biophysical models and hybrid species distribution models, we found: (i) thermal safety margin is larger at high latitudes and is predicted to decrease under climate warming for lizards at all latitudes; (ii) climate warming will speed up embryonic development and increase annual activity time of adult lizards, but will exacerbate water loss of adults across all latitudes; and (iii) species across latitudes are predicted to experience habitat contraction under climate warming due to different limitations—tropical and subtropical species are vulnerable due to increased extremely high temperatures, whereas temperate species are vulnerable due to both extremely high temperatures and increased water loss. This study provides a comprehensive understanding of the vulnerability of species from different latitudinal regions to climate warming in ectotherms, and also highlights the importance of integrating environmental factors, behaviour, physiology and life-history responses in predicting the risk of species to climate warming.

## 1. Introduction

Anthropogenic climate warming has imposed a massive threat to global biodiversity [1–3]. Due to differences in the magnitude of climate warming and species sensitivities to such perturbations [4,5], the impact of climate warming on species varies across latitudes [2,6,7]. A comprehensive understanding of the vulnerabilities of species from different latitudinal regions to climate warming is critical for future conservation planning [8].

Controversy over the vulnerabilities of species to climate warming across latitudes has spanned decades [4,7,9,10]. Early studies predicted that species at mid- to high latitudes, especially in the Northern Hemisphere, would be more vulnerable [7,11,12] based on faster rates of warming at high latitudes [11,13]. Later studies which considered species' physiology (i.e. metabolic rate and heat tolerance) proposed the opposite: that low-latitude species are likely to be more vulnerable than high-latitude counterparts under climate warming [4,8,14,15]. However, the most recent re-evaluation of the thermal safety margin (TSM; the difference between maximum operative temperature and species’ critical thermal maximum [8]) in terrestrial insects indicated that tropical and temperate species might face a similar threat after accounting for seasonal activity times [16]. Therefore, conclusions about the latitudinal variation of species' vulnerability to climate warming have ‘evolved’ over time as more critical parameters were considered in the analyses. More recently, parameters including physiological, behavioural and life-history responses are being increasingly used when evaluating species vulnerabilities [10,17,18].

An integrative consideration of species' behavioural and physiological responses is essential to gain a more comprehensive understanding of species' vulnerability to climate warming from different latitudes [19,20]. First, the TSM reflects the potential tolerance of animals to the external thermal environment, which provides an easy but effective way of estimating vulnerabilities to warming temperatures [4,8,21]. Second, as a consequence of behavioural thermoregulation, activity time has been documented as a critical factor in predicting the vulnerability of animals to warming [22,23]. This is because insufficient activity time may limit overall energy and water intake and inhibit development, growth and reproduction in ectotherms [22,24]. Third, metabolic rate is an essential parameter for predicting extinction risk because it potentially affects individuals' net energetic gain [9], growth [25] and even lifespan [24]. Fourth, corresponding to changes in precipitation wrought by climate warming, water loss affects energy and water dynamics and therefore determines the vulnerability of ectotherms to warming in synergy with temperature [17,26]; as increased water loss has been increasingly found to directly induce population collapse, especially in dry areas [27,28].

On the basis of behavioural and physiological responses, species distribution range shifts are one of the most reliable indicators of the risk of extinction and are widely employed to assess the impact of warming on species [29–32]. Species distribution models (SDMs) with both environmental and physiological predictors (hereafter hybrid-SDMs) provide robust and useful insights into ‘where’ and ‘why’ species will persist or go extinct under warming [33], as it considers not only the environmental niche but also a species' behavioural and physiological responses [34–37].

Species with large distributions across a wide latitudinal span, with well-known behavioural, physiological and life-history traits are required for evaluating the vulnerabilities of species from different latitudes to climate warming. *Takydromus* lizards (commonly named grass lizards) are a genus of small lacertid lizards (snout–vent length [SVL] less than 70 mm), containing 23 recognized species that are widely distributed in eastern, southern and southeastern Asia [38–40]. In China, *Takydromus* lizards are distributed along the eastern coast spanning a wide latitudinal gradient from tropical to temperate areas (18°09′–53°35′ N) and have been the subject of intensive research on their behavioural, physiological and life-history traits [41–44]. Therefore, *Takydromus* lizards from different latitudinal regions constitute a great study system for investigating the vulnerabilities of species from different latitudes to climate warming, with integrative considerations of behavioural, physiological and life-history traits.

In this study, we used *Takydromus* lizards from tropical to temperate areas in China as our model system to investigate the vulnerabilities of species from different latitudinal regions to climate warming. First, we estimated TSM and fitness-related traits with biophysical models for each species, and then integrated these fitness-related traits in hybrid-SDM to predict species’ range shifts under climate warming. Here, we propose that the *Takydromus* lizards from tropical regions would be more vulnerable to climate warming than their counterparts from medium and high latitudes, due to their reduced TSM, depressed fitness-related traits and contraction of the suitable distribution range.

## 2. Materials and methods

### (a) Materials

#### (i) Biological traits

**Figure 1.**
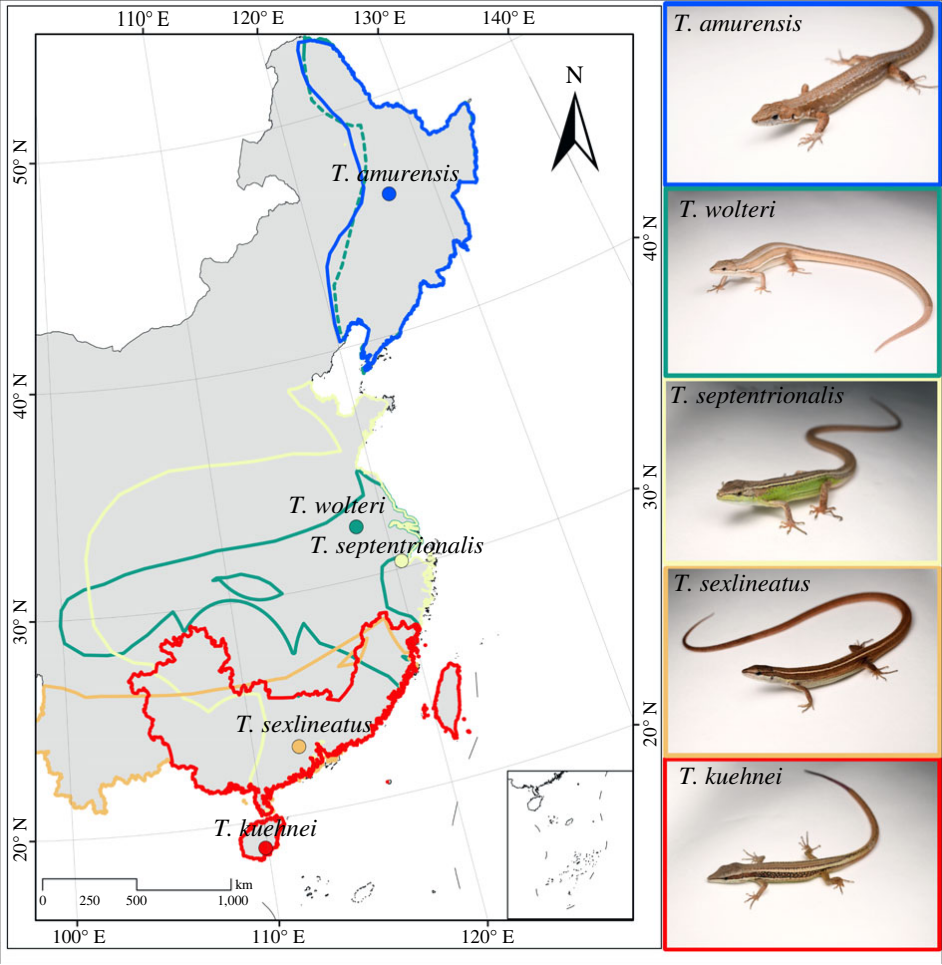
Distribution ranges of five *Takydromus* lizard species and the locations of the populations sampled for behavioural, physiological and life-history responses to climate warming. Different colours in outlines indicate different species in the map and species photographs. (Online version in colour.)

We selected five species of *Takydromus* lizards with a wide geographical distribution across tropical, subtropical and temperate areas of China as our study system (figure 1): *T. amurensis* from temperate areas, *T. wolteri* and *T. septentrionalis* from subtropical areas, and *T. sexlineatus* and *T. kuehnei* from tropical areas [40,45–47]. We collected data for body mass, critical thermal maximum (CT_max_), critical thermal minimum (CT_min_) and selected body temperatures for adults from literature [41–44]. To obtain temperature data for foraging, basking and leaving their retreat, we recorded active body temperatures in the field for five species across the active seasons (electronic supplementary material, table S1, see more details in electronic supplementary material, Methods and data S1). According to our measurement, the minimum field body temperatures recorded during the active season were 16.18–18.29°C in all five species; we set the average temperature of 17°C as the leaving retreat temperature accordingly. Further, after a quick test, we found that the leaving retreat temperature within the range of 16.18–18.29°C as input in the biophysical model (NicheMapR) does not influence the model result. We also collected data for embryonic development from the previous studies [10,48–55] (see details in electronic supplementary material, table S2 and data S1). It is noteworthy that *T. wolteri* has a disjunct distribution. We only collected the physiological parameters from the southern population, which is far away from the northern population. Therefore, in our further analysis, we only assess the impact of climate change on its southern population.

#### (ii) Microclimate data

We used the microclimate model in the NicheMapR package in R 3.6.2 to extract hourly estimates of microclimates. The microclimate model (implemented in the ‘micro_global’ function) provides hourly estimates of solar and infrared radiation, aboveground air temperature, wind velocity, relative humidity at the animal's height and soil temperature profiles (see more details in [56,57]). All current climate layers used by the NicheMapR are from published protocols [58], with a resolution of 10 arc-minutes. To obtain estimations of the microclimates under 2050 and 2070 climate forecasts, we downloaded monthly maximum and minimum temperature and precipitation from the WorldClim dataset in 2050 (2041–2060) and 2070 (2061–2080) with a resolution of 10 arc-minutes. We considered three global circulation models (GCMs: BCC-CSM1-1, CNRM-CM5 and MIROC-ESM, see details in [59–61]) and two emission scenarios (Representative Concentration Pathways: RCP 4.5 and RCP 8.5) representing mild and extreme predicted impacts of warming. Following a widely used approach in climate projections generated from different GCMs, we created an ensemble projection by averaging projections of the three GCMs [62–67]. We downloaded the altitude layer from open-source GTOPO30 (https://earthexplorer.usgs.gov/), derived slope and aspect layers from the altitude layer using ArcGIS v10.5, and then resampled all three layers to a resolution of 10 arc-minutes using bilinear interpolation. We validated the microclimate model by using air temperature from ERA5 (https://www.ecmwf.int/en/forecasts/datasets/reanalysis-datasets/era5) to predict air temperatures at a specific height (1 cm) at the sample site and then compared the predicted values with temperature recorded by data loggers (iButton, https://www.ibuttonlink.com/collections/ibuttons). The results show that the microclimate model performs well in predicting air temperatures (*R* = 0.797, RMSE = 6.275; electronic supplementary material, figure S1).

#### (iii) Macroclimate data

We used five bioclimate variables to describe the macroclimate conditions encountered by lizards: annual mean temperature (bio1), maximum temperature of the warmest month (bio5), minimum temperature of the coldest month (bio6), annual mean precipitation (bio12) and mean sum of precipitation in the warmest quarter (bio18). We chose these variables because they reflect two primary properties of the climate—energy and water—that have known roles in imposing constraints on species distribution due to widely shared physiological limitations (following [68]). To standardize the climate data for use in our biophysical models and hybrid-SDMs, we obtained current monthly climate layers (maximum and minimum temperature, precipitation) from [58] and then generated bioclimate layers with the ‘biovars’ function in the ‘dismo’ package. We obtained future bioclimate layers (2050 and 2070) from the WorldClim dataset [69] (www.worldclim.org), using the same global circulation models and emission scenarios in the microclimate model and biophysical model.

#### (iv) Species occurrence records

We collected occurrence records of the five grass lizard species from 185 published literature and Nature Reserve investigation reports between 1990 and 2019 (see electronic supplementary material, file S1) and the Global Biodiversity Information Facility (https://www.gbif.org/; accessed in June 2020). To avoid errors arising from the occurrence records, we removed any data from outside of the species' spatial distribution range [70]. We also used the ‘CoordinateCleaner’ package [71] in R to remove records assigned to the capitals, institutes and museums. To avoid potential spatial autocorrelation among occurrence records, we used the ‘spThin’ package [72] to thin the records with 10 arc-minutes [73].

### (b) Methods

#### (i) Thermal safety margin and fitness-related traits

Using this microclimate data along with morphological and physiological traits of each species as inputs, we ran a biophysical model for ectotherms using the ‘ectotherm’ function in NicheMapR. This model integrates hourly estimates of microclimate and species traits to calculate operative temperatures (T_e_) in microhabitats, species' activity time, metabolic rate and water loss based on the processes of heat and water exchange. To run the biophysical model for future climates, we replaced the current climate layer with climate layers of future climates in the microclimate model (‘micro_global’ function).

In biophysical models, we used the median value of body mass for each species as input for body size and set their midpoints at 1 cm above the ground and only diurnal activity, bounded within the maximum and minimum (75% and 25% quantile in this study) temperature for activity. When operative temperatures above ground fell outside of these temperatures for activity, animals were simulated to burrow underground to a depth where the temperature corresponds to their preferred temperature, as obtained from laboratory experiments. The leaving retreat temperature was set as the minimum body temperature (electronic supplementary material, table S1). From these measurements, we calculated the TSM with the equation of TSM = CT_max_ – T_e-max_, where T_e-max_ was the maximum value of hourly operative temperatures (T_e_) in the entire year. Activity time was calculated as the number of hours that the lizard was predicted to be active. Metabolic rate was represented by an allometric function based on oxygen consumption rate (ml h^−1^) [74], and we used the maximum oxygen consumption rate as a metric related to fitness. For water loss, we calculated the sum of all components of water loss (i.e. respiratory water loss, cutaneous water loss and ocular water loss) in biophysical models and took the maximum value as a metric. All input parameters of lizards' biological traits are found in the electronic supplementary material, table S1, with the remaining parameters set to default (electronic supplementary material, data S2). We also developed an embryo incubation model based on the incubation period and hatching success at different temperatures (electronic supplementary material, table S2) and from that calculated incubation period and the number of hours that are suitable for embryonic development over a year (hereafter time window) based on microclimate data.

#### (ii) Species distribution range

Hybrid-SDMs were constructed for each species using occurrence records and predictors. The predictors contained bioclimate variables (i.e. bio1, bio5, bio6, bio12 and bio18; electronic supplementary material, figures S2 and S3) and outputs from biophysical models (i.e. activity time, metabolic rate, water loss, incubation period and time window suitable for embryonic development). We used five modelling algorithms: generalized linear model (GLM), generalized boosted regression models (GBM), maximum entropy (MaxEnt), random forest (RF) and support vector machines (SVM), and generated pseudo absences using the ‘eRandom’ method [75]. We then used a 70% random sample of initial data as training data and evaluated them against the remaining 30% [76], repeated five times for each correlative niche model algorithm. We evaluated model performance using the area under the receiver operating characteristic curve (AUC [77]) and true skill statistics (TSS [78]). We only kept models with a TSS value higher than 0.6 [79] and applied the TSS method to weighted models to build ensemble models to obtain a species habitat suitability map at 10 arc-minutes resolution [80–82]. Habitat suitability is defined in terms of the capacity of a given habitat to support a selected species based on the biophysical and bioclimate variables measured. These procedures use an index that ranges from 0, for unsuitable habitat, to 1 for optimal habitat [83]. We classified binary maps (presence/absence) with the threshold by maximizing the TSS value [84] from the ensemble forecasts for the current, 2050 and 2070 periods to represent the species distribution range.

Because of the limited capacity for dispersal in lizards [85,86], we limited species study areas to their current distribution range, and clipped TSM, activity time, metabolic rate, water loss, incubation period, time for embryonic development (time window), habitat suitability and distribution range into their current species distribution for further analyses.

#### (iii) Variable contribution to the change of suitability

We analysed the contribution of physiological and climate variables to changes in habitat suitability, because habitat suitability can be directly predicted by physiological and climate variables. We averaged the changes for each variable across all grids of current distributional ranges using hybrid-SDMs and calculated the corresponding change in habitat suitability for each species induced by that variable alone (other variables were set to their mean values) using the response curves from the hybrid-SDMs. Finally, we used the ‘getVarImp’ function in the ‘sdm’ package to obtain the value of each predictor from five niche model algorithms (GLM, GBM, MaxEnt, RF and SVM), following our previously published protocols [67], see a complete flow chart of methods in the electronic supplementary material, figure S4). We only show the results based on the RCP 4.5 emission scenario in the text and put the related results based on the RCP 8.5 scenario in the electronic supplementary material.

## 3. Results

### (a) Thermal tolerance and thermal safety margin

**Figure 2.**
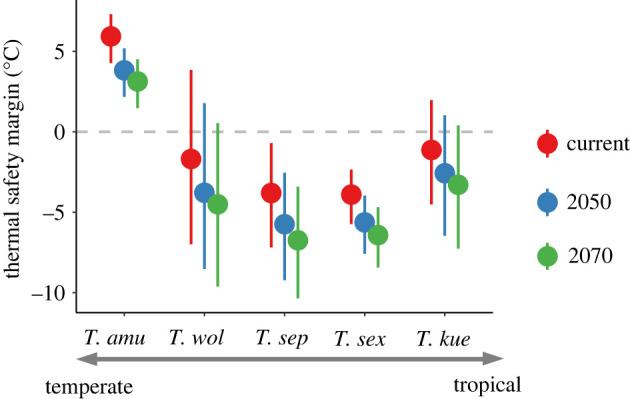
TSM for five grass lizard (*Takydromus*) species across latitudes. Red, blue and green spots indicate the TSM under current, 2050 and 2070 situations. The species are listed from temperate to tropical areas on the *x*-axis. Error bars represent s.d. Because of the same trends of future climate, we only show the results of the RCP 4.5 emission scenario in the text. (Online version in colour.)

For all periods we considered (current, 2050 and 2070), the TSM was larger for lizards from temperate (*T. amurensis*) than from tropical (*T. kuehnei* and *T. sexlineatus*) and subtropical areas (*T. septentrionalis*) (figure 2; Wilcoxon test, all *p* < 0.001). Under climate warming, TSM gradually decreases in the future, which suggests an increased risk of experiencing heat stress, especially for tropical (*T. kuehnei* and *T. sexlineatus*; Wilcoxon test, both *p* < 0.001) and subtropical species (*T. septentrionalis* and *T. wolteri*; Wilcoxon test, both *p* < 0.001). We observed same patterns under the RCP 8.5 scenario (electronic supplementary material, figure S5).

### (b) Fitness-related responses

**Figure 3.**
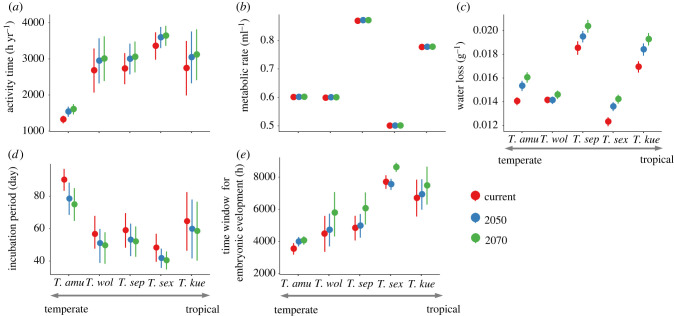
Fitness-related traits for five grass lizard (*Takydromus*) species across latitudes in the current climate (red dots), and predictions for 2050 (blue dots) and 2070 (green dots). (*a*) Activity time, (*b*) metabolic rate, (*c*) water loss, (*d*) incubation period and (*e*) time window for successful embryonic development. Error bars represent s.d. Because of the same trends of future climates, we only show the RCP 4.5 emission scenario results in the text. (Online version in colour.)

For adult *Takydromus* lizards, activity time increased more in tropical species (tropical versus other regions: 273 versus 260 h yr^−1^ in 2050 and 336 versus 315 h yr^−1^; Wilcoxon test, both *p* < 0.05) and was predicted to increase under climate warming for all species (figure 3*a*). The metabolic rate varied among species and would change slightly in all species under climate warming (±0.0037 ml h^−1^ both for 2050 and 2070; figure 3*b*). By contrast, water loss is complex. Higher levels of water loss were found under the present climate in tropical (*T. kuehnei*) and subtropical species (*T. septentrionalis* [0.018 g h^−1^ in average] compared to the other three species (0.013 g h^−1^ in average; Wilcoxon test, *Z* = 82.6, *p* < 0.001). Water loss was predicted to increase under climate warming for all five species, even for temperate species *T. amurensis*, which had less water loss under current conditions. By contrast, the smallest increase in water loss was predicted for *T. wolteri* (*T. wolteri* versus other four species: 0.0000 versus 0.0011 g h^−1^ in 2050 and 0.0004 versus 0.0019 g h^−1^ in 2070; Wilcoxon test, both *p* < 0.001; figure 3*c*). Currently, embryos of temperate species (i.e. high latitude) required more days for development than others (*T. amurensis* versus other four species: 107 versus 63 days; Wilcoxon test, *Z* = 53.4, *p* < 0.001; figure 3*d*). Further, the incubation period was predicted to decrease under climate warming in all species (figure 3*d*). The time window suitable for incubation increased towards low latitudes currently and was predicted to increase under climate warming in all five species (increase 138 h and 1032 h in 2050 and 2070; figure 3*e*). In summary, all five species were predicted to benefit from climate warming because of increased activity time, reduced incubation period and increased time window suitable for successful embryonic development. However, our models predicted that all five species would be at risk because of suffering increased water loss under climate warming. Similar patterns were also found under the RCP 8.5 scenario (electronic supplementary material, figure S6).

### (c) Change in suitable distribution range

**Figure 4.**
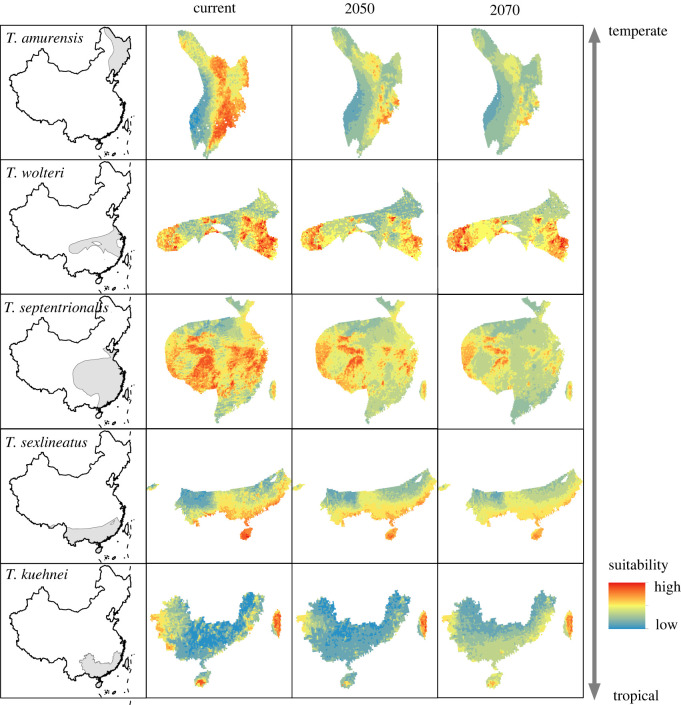
Spatial distribution and habitat suitability of five grass lizard (*Takydromus*) species in the current climate, and predictions for 2050 and 2070. The first two columns indicate the species distribution in China derived from spatial distribution maps (indicated by [70]). The third and fourth columns indicate the habitat suitability in 2050 and 2070 under climate warming from Hybrid-SDMs for each species. The colour indicates the suitability. The list of species on the *y*-axis indicates the latitudinal areas of the species. Because of the same trends of future climate, we only show the RCP 4.5 emission scenario results in text. (Online version in colour.)

**Figure 5.**
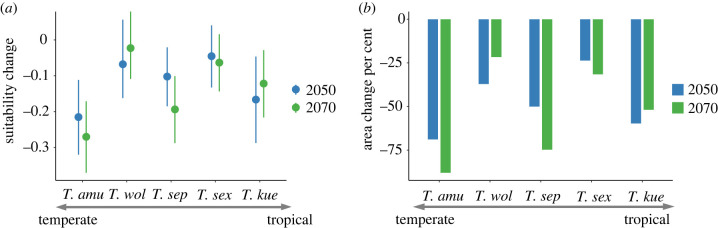
Change of habitat suitability (*a*) and per cent area (*b*) for five grass lizard (*Takydromus*) species across latitudes under current and 2050 and 2070 climate warming. Blue and green spots (*a*) and bars (*b*) indicate the traits of 2050 and 2070, respectively. Error bars in (*a*) represent s.d. Because of the same trends of future climate, we only show the RCP 4.5 emission scenario results in the text. (Online version in colour.)

According to AUC and TSS values, the hybrid-SDMs performed well in this study (mean AUC = 0.883, 95% CI = 0.874–0.892; mean TSS = 0.758, 95% CI = 0.743–0.774; electronic supplementary material, table S3). Habitat suitability, and therefore distribution range, is predicted to decrease in future for all five species (figures 4 and 5) under climate change. *T. amurensis* from the temperate area will experience the greatest decrease in habitat suitability (decrease 0.225 and 0.372 on average for 2050 and 2070, respectively). *T. wolteri* from subtropical areas and *T. sexlineatus* from tropical areas are predicted to experience the least decrease in habitat suitability (decrease 0.08 and 0.05 on average for 2050, 0.03 and 0.06 for 2070, respectively). This difference still held even when we converted habitat suitability to a binary map (presence/absence) and calculated the change in suitable habitat area. The net habitat loss is predicted to be the greatest for *T. amurensis* (68.9%–88.0%; temperate species), and the least for *T. wolteri* (21.7%–37.6%; subtropical species) and *T. sexlineatus* (23.6%–31.5%; tropical species). The results under RCP 8.5 scenario see electronic supplementary material, figure S7.

### (d) Variable contribution to the change of suitability

**Figure 6.**
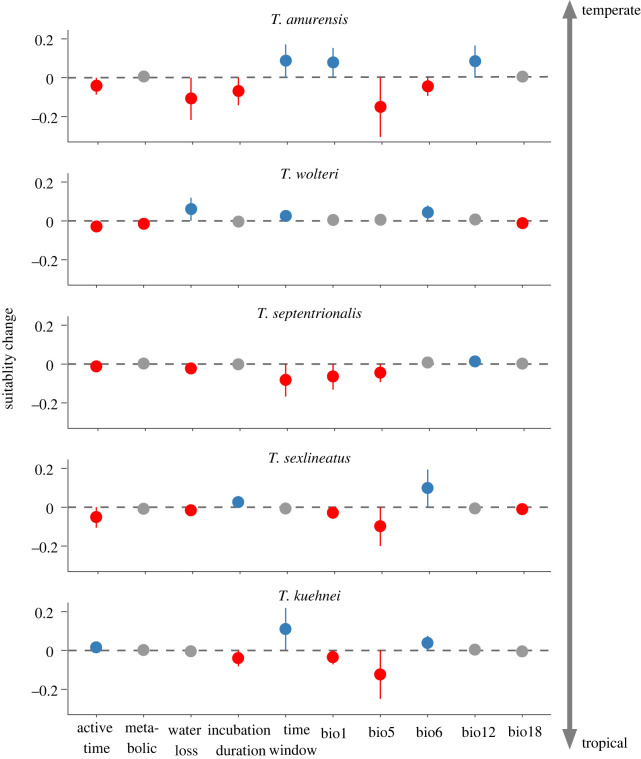
Changes of habitat suitability in 2070 caused by fitness-related and bioclimate variables under a climate warming scenario (RCP 4.5) in five grass lizard (*Takydromus*) species. Blue, grey and red spots indicate increased, unchanged and decreased suitability by a threshold of 0.01 for average change, respectively. Error bars represent s.d. Suitability change is a value that measures the difference in habitat suitability index at present and under climate change. (Online version in colour.)

The differences for changes in habitat suitability caused by predictors (representing variable contributions) were consistent for 2050 (electronic supplementary material, figure S8) and 2070 (figure 6). The predicted decrease in habitat suitability for tropical and subtropical species (*T. kuehnei*, *T. sexlineatus* and *T. septentrionalis*) was due to the rise in annual mean temperature (bio1) and maximum temperature of the warmest month (bio5; figure 6). By contrast, the predicted decrease in habitat suitability for temperate species (*T. amurensis*) was mainly due to maximum temperature of the warmest month (bio5) and the increased water loss. Similar results were also found under the RCP 8.5 scenario (electronic supplementary material, figure S9).

## 4. Discussion

Evaluating the vulnerabilities of animals to climate warming can provide comprehensive management perspectives for preparing future conservation plans [10,18,87]. In this study, we used biophysical models and hybrid-SDMs integrating behavioural, physiological and life-history responses and distribution range change of the widespread *Takydromus* genus across latitudes to better understand the vulnerability of species to climate warming. Our results showed that both tropical and temperate species are vulnerable to climate warming, albeit for different reasons. We found that the greatest threat of climate warming for tropical species was due to increasing temperatures. Surprisingly, we found that temperate species are also highly vulnerable to warming due to increasing temperatures and greater rates of water loss. Our results provide novel insights into current understanding of the species' vulnerability to climate warming at different latitudes.

The results from our TSM analyses suggest that tropical (i.e. *T. kuehnei* and *T. sexlineatus*) and subtropical species (*T. septentrionalis* and *T. wolteri*) have relatively smaller TSMs than temperate species (*T. amurensis*) under warming (figures 2 and 5). This is consistent with previous findings that tropical ectotherms are more vulnerable to climate warming due to their small TSMs [4,8]. Tropical ectotherms currently experience temperatures close to their thermal tolerance. As a result, even a small increase in temperature may push species toward or even beyond their heat tolerance thresholds, ultimately leading to precipitous declines in performance and fitness [4,88]. Physiological responses and distribution area predictions from our study further revealed that both mean annual temperatures (bio1) and extremely high temperature (bio5, maximum temperature in warmest month) impose a critical threat to species from tropical areas (*T. kuehnei* and *T. sexlineatus*; figure 6). Moreover, in accordance with findings from recent studies showing that sites with local extinctions had significantly lower mean annual temperatures but larger increases in maximum yearly temperatures [89,90], we found that temperature extremes (bio5) rather than mean temperatures (bio1) were significant contributors to loss of habitat suitability in our model system under warming (figure 6).

Interestingly, we find that tropical (*T. kuehnei* and *T. sexlineatus*) and subtropical (*T. septentrionalis* and *T. wolteri*) species are not equally vulnerable to warming. In fact, tropical species of *T. sexlineatus* and subtropical species of *T. wolteri* are less vulnerable compared with tropical *T. kuehnei* and subtropical *T. septentrionalis* (figure 5). This is likely to be due to between-species differences in thermal biology traits. For example, *T. sexlineatus* has a longer annual activity time, shorter incubation period and longer time window suitable for embryonic development than the other two species (figures 2 and 3) and thus may be less vulnerable to warming. Similarly, several species of tropical *Anolis* lizards are predicted to respond differently to warming despite the fact that they occupy structurally similar forest habitats, with one species experiencing reduced activity times as a result of warming, while the other two species not [91]. Additionally, interspecific divergence in preferred microhabitat characteristics also may contribute to differences in species’ vulnerability. For example, xeric populations are more vulnerable to warming than mesic populations in tropical Caribbean lizards [92]. Similarly, *T. kuehnei* from high elevation (i.e. greater than 900 m) has not been severely threatened by climate warming [93]. Apart from these kinds of large-scale geographical (e.g. latitudinal or altitudinal) variations in species vulnerabilities to climate warming, our study highlights the importance of assessing species vulnerability at a local scale using high-resolution climate data and species traits to fully evaluate the biological impact of warming.

Previous research predicted temperate lizards would be at lower risk of climate warming than tropical species, because temperate species are exposed to temperatures well below their thermal optima, and thus may be at reduced risk, or even benefit from increasing temperatures [4,14,94]. By contrast, we found that temperate lizards would be also highly vulnerable to warming because of increased water loss and rise of heat stress by extreme high temperature under climate warming. Our biophysical model predicts that *Takydromus* lizards will benefit from climate warming by having increased the time window (the number of hours that are suitable) for embryonic development (figures 3 and 6). Conversely, increased water loss can potentially override those benefits for temperate species, driving a decrease in habitat suitability and inducing greater vulnerabilities to climate warming. Similarly, the response of the Australian sleepy lizards (*Tiliqua rugosa*) to climate warming depends on future patterns of rainfall [24]. In many physiology-based studies, precipitation and water loss have been ignored when assessing warming effects [8,9,95–98]. Our study has revealed that the vulnerability of species to climate warming may be underestimated if the organismal water balance of species is not considered, even in terrestrial vertebrates. The effects of increasing temperature on organismal water balance are likely to drive some population declines, because more water will be lost as a cooling cost [27,28]. Although ectothermic vertebrates may be able to prevent excessive dehydration resulting in lethargy and death via spending more time retreating or seeking water [99], such changes may sacrifice opportunities for other behaviours such as basking and feeding, which may also influence survival and push species to be under greater risk of climate warming [96,100].

In summary, we found tropical and temperate *Takydromus* lizards would be vulnerable to climate warming due to extremely high temperatures and substantial water loss, respectively. This study inspires more research on latitudinal differences with the consideration of integrating the behavioural, physiological and life-history responses of species. As the high demand for parameters of species, we only used five *Takydromus* species, which were well documented in their thermal physiology. This may limit the conclusion of the latitudinal pattern of the vulnerabilities of species to climate warming. It is also notable that we used the parameters from one population to model the entire distribution for all species. More research investigating the behavioural, physiological and life-history responses of other lineages and more populations is needed to conclude the generality of a latitudinal pattern of the vulnerabilities of species to climate warming. Moreover, our results reinforce the benefits of integrative mechanistic models (e.g. the biophysical model and hybrid-SDMs) in predicting the impact of climate warming on biodiversity, in which behavioural, physiological and life-history traits are considered in concert with macro- and micro-climatic data [17,101].

## Data Availability

All data supporting this article are available online at Dryad: https://doi.org/10.5061/dryad.1g1jwsv0g [102]. Electronic supplementary material is available online [103].
